# Tissue-specific differential induction of duplicated fatty acid-binding protein genes by the peroxisome proliferator, clofibrate, in zebrafish (*Danio rerio*)

**DOI:** 10.1186/1471-2148-12-112

**Published:** 2012-07-09

**Authors:** Ananda B Venkatachalam, Santosh P Lall, Eileen M Denovan-Wright, Jonathan M Wright

**Affiliations:** 1 Department of Biology, Dalhousie University, Halifax, NS, B3H 4R2, Canada; 2 National Research Council of Canada, Institute of Marine Biosciences, Halifax, NS, B3H 3Z1, Canada; 3 Department of Pharmacology, Dalhousie University, Halifax, NS, B3H 4R2, Canada

## Abstract

**Background:**

Force, Lynch and Conery proposed the duplication-degeneration-complementation (DDC) model in which partitioning of ancestral functions (subfunctionalization) and acquisition of novel functions (neofunctionalization) were the two primary mechanisms for the retention of duplicated genes. The DDC model was tested by analyzing the transcriptional induction of the duplicated fatty acid-binding protein (*fabp*) genes by clofibrate in zebrafish. Clofibrate is a specific ligand of the peroxisome proliferator-activated receptor (PPAR); it activates PPAR which then binds to a peroxisome proliferator response element (PPRE) to induce the transcriptional initiation of genes primarily involved in lipid homeostasis. Zebrafish was chosen as our model organism as it has many duplicated genes owing to a whole genome duplication (WGD) event that occurred ~230-400 million years ago in the teleost fish lineage. We assayed the steady-state levels of *fabp* mRNA and heterogeneous nuclear RNA (hnRNA) transcripts in liver, intestine, muscle, brain and heart for four sets of duplicated *fabp* genes, *fabp1a/fabp1b.1/fabp1b.2, fabp7a/fabp7b, fabp10a/fabp10b* and *fabp11a/fabp11b* in zebrafish fed different concentrations of clofibrate.

**Result:**

Electron microscopy showed an increase in the number of peroxisomes and mitochondria in liver and heart, respectively, in zebrafish fed clofibrate. Clofibrate also increased the steady-state level of *acox1* mRNA and hnRNA transcripts in different tissues, a gene with a functional PPRE. These results demonstrate that zebrafish is responsive to clofibrate, unlike some other fishes. The levels of *fabp* mRNA and hnRNA transcripts for the four sets of duplicated *fabp* genes was determined by reverse transcription, quantitative polymerase chain reaction (RT-qPCR). The level of hnRNA coded by a gene is an indirect estimate of the rate of transcriptional initiation of that gene. Clofibrate increased the steady-state level of *fabp* mRNAs and hnRNAs for both the duplicated copies of *fabp1a/fabp1b.1,* and *fabp7a/fabp7b*, but in different tissues. Clofibrate also increased the steady-state level of *fabp10a* and *fabp11a* mRNAs and hnRNAs in liver*,* but not for *fabp10b* and *fabp11b*.

**Conclusion:**

Some duplicated *fabp* genes have, most likely, retained PPREs, but induction by clofibrate is over-ridden by an, as yet, unknown tissue-specific mechanism(s). Regardless of the tissue-specific mechanism(s), transcriptional control of duplicated zebrafish *fabp* genes by clofibrate has markedly diverged since the WGD event.

## Background

In 1970, Ohno [1] proposed that duplication of individual genes, chromosomal segments or whole genomes plays an important role in genome evolution, provides for increasing organismal complexity and contributes to morphological diversification among vertebrates [2-4]. The role of gene duplicates in generating morphological and functional diversity has been discussed by various researchers (see [5] and references therein). To our knowledge, Ohno [1] was the first to suggest possible fates for duplicated genes by the process of either nonfunctionalization or neofunctionalization. Nonfunctionalization of a duplicated gene occurs due to deleterious mutations accumulating in the protein coding region, leading to gene silencing and subsequent loss of one of the duplicate genes from the genome. Ohno [1] further argued that nonfunctionalization is the common fate of a duplicated gene. Neofunctionalization results from mutations in the protein coding region that gives rise to a novel function for a gene product. If this novel function benefits the organism, the gene will be retained in the genome. With complete genomic DNA sequences becoming increasingly available, it is apparent that a greater proportion of gene duplicates are preserved in genomes than that predicted by Ohno’s model [6]. In light of these observations, Force *et al.*[6], subsequently elaborated by Lynch and Conery [7], proposed the duplication-degeneration-complementation (DDC) model. In the DDC model, subfunctionalization is the process by which the functions of the ancestral gene are subdivided between the duplicated genes. Subfunctionalization in the DDC model was proposed as an alternative mechanism to Ohno’s neofunctionalization [1] to explain the high retention rate of duplicated genes in the genome. Force *et al.*[6], however, did not exclude neofunctionalization, in which one of the duplicated genes acquires a novel function. In the DDC model, subfunctionalization and neofunctionalization occur by either loss or gain of *cis*-regulatory elements in the promoters of the duplicated genes.

Fatty acid-binding protein *(FABP)* genes belong to the multigene family of intracellular lipid-binding protein (*iLBP*) genes that also includes the cellular retinol-binding protein (*CRBP*) and the cellular retinoic acid-binding protein (*CRABP*) genes [8-12]. To date, eighteen paralogous *iLBP* genes, including 12 *FABPs*, 4 *CRBPs* and 2 *CRABPs* have been identified in the animal kingdom. No *FABP* genes have been found in plants or fungi, leading Schaap *et al.*[9] to suggest that the first *FABP* gene emerged after the divergence of animals from plants, some 930–1000 million years ago (mya). About 230–400 mya, the *iLBP* multigene family was further augmented in teleost fishes by a whole genome duplication (WGD) event early in teleost fish lineage [4,13-17]. Based on complementary DNA (cDNA) sequence, gene structure, conserved gene synteny with their mammalian, avian and fish orthologs, and spatio-temporal patterns of expression, we have characterized 12 zebrafish *fabp* genes [18-30]. Of these 12 zebrafish *fabp* genes, eight (four pairs) *fabp1a/fabp1b, fabp7a/fabp7bfabp10a/fabp10b* and *fabp11a/fabp11b* arose as a result of the teleost fish-specific WGD [23,25,28,29]. One pair of duplicated genes, *fabp1b.1 and fabp1b.2*, is tandemly arrayed on chromosome 8 separated by 3.8 kb of DNA [30]. This duplication, subsequent to the WGD early in the fish lineage, is presumably the result of unequal crossing-over between homologous chromosomes during meiosis. The total number of duplicated genes retained in the zebrafish genome following the WGD event is estimated to be 14-30% [31,32]. Surprisingly, 73% of the duplicated *fabp* genes have been retained in the zebrafish genome. Only three zebrafish *fabp* genes exist as single copies, *fabp2fabp3* and *fabp6*. Originally, FABPs were named according to their initial tissue of isolation. This nomenclature has become increasingly confusing as some tissues contain more than one FABP, and some FABPs are found in many tissues. As such, we have chosen to use in this paper the nomenclature proposed by Hertzel and Bernlohr [33]*e.g.,* FABP1, FABP2, *etc.* Although different *FABP* genes exhibit distinct, but sometimes overlapping, tissue-specific patterns of expression, the tertiary structure of *FABP* genes and their genomic organization are highly conserved [12,34,35]. Almost all *FABP*/*fabp* genes, with the exception of the *FABP3* gene in desert locust [36], the *fabp1a* gene from zebrafish [25] and *fabp11a* gene from medaka [37], consist of four exons of comparable coding capacity separated by three introns of varying sizes between paralogous and orthologous *FABP*/*fabp* genes in different species [8,9,38-40]. Despite extensive studies on the structure of FABPs, binding properties and *in vitro* lipid transfer mechanisms, their precise physiological role remains elusive. However, several studies [reviewed in 12] have implicated FABPs in myriad cellular processes that include: (1) binding and sequestering of long-chain fatty acids, bile salts and other hydrophobic ligands; (2) transport of these ligands to intracellular compartments for metabolism and energy production; (3) interaction with other enzyme systems and transport proteins; and (4) transport of fatty acids (FAs) to the nucleus to regulate gene transcription *via* activation of the nuclear receptors, the peroxisome proliferator-activated receptors (PPARs) (see [41] and references therein). Currently, our knowledge of the regulatory elements controlling the expression of the *FABP* genes is limited and based mainly on studies of mammalian *FABP* genes and one *FABP* gene in desert locust [42-46]. Her *et al.*[47,48] cloned the 5' upstream regions, including the basal promoters, of the zebrafish *fabp10a* and *fabp2* genes. They identified a 435 base pairs (bp) region with two distinct liver regulatory elements in the liver-basic fatty acid-binding protein (*fabp10a*) gene, which is sufficient to modulate liver regional expression in transgenic zebrafish [47]. A 192 bp region was identified in the 5' upstream region of the intestinal-type fatty acid-binding protein (*fabp2*) gene sufficient to direct intestine-specific expression in zebrafish larval development [48]. Neither of these studies provided insight into why both duplicated *fabp10* genes, *fabp10a* and *fabp10b,* were retained in the zebrafish genome, or why a copy of the duplicated *fabp2* gene was lost from the zebrafish genome following the WGD in the teleost fishes.

In previous studies, we have shown that transcriptional initiation of only one copy in each of three sets of duplicated *fabp* genes of zebrafish, *fabp1a/fabp1b.1/fabp1b.2, fabp7a/fabp7b* and *fabp11a/fabp11b*, is modulated by dietary FAs in a given tissue [49]. Since FAs are known to be ligands of PPARs that leads to transcriptional up-regulation of target genes, we anticipated that the transcriptional modulation of *fabp* genes in various tissues of zebrafish fed different FAs might be mediated by PPARs. The goal of the present research was, therefore, to investigate whether the duplicated *fabp* genes in zebrafish are differentially regulated by PPAR, by using clofibrate, a PPAR agonist. Clofibrate has been used extensively to investigate the regulation of gene transcription in vertebrates, owing to its specific binding with PPARα, and to a lesser extent to PPARγ, and its effect on the transcription of specific genes involved in lipid metabolism [50-57]. We assayed the steady-state levels of *fabp* mRNA and heterogeneous nuclear RNA (hnRNA) transcripts for four sets of duplicated *fabp* genes, *fabp1a/fabp1b.1/fabp1b.2, fabp7a/fabp7b, fabp10a/fabp10b* and *fabp11a/fabp11b* in zebrafish fed different concentrations of clofibrate to determine if clofibrate induced transcriptional initiation of only one of a pair of duplicated *fabp* genes. We show here, however, that clofibrate induced the transcriptional initiation of both pairs of some duplicated *fabp* genes in zebrafish, but the induction is differentially regulated by an, as yet, unknown tissue-specific mechanism(s).

## Results and discussion

### Zebrafish is responsive to the peroxisome proliferator, clofibrate

**Figure 1:**
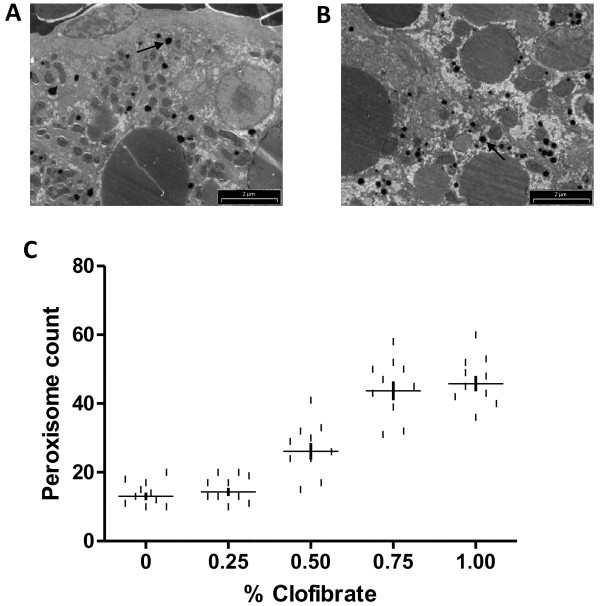
**Electron micrographs of hepatocytes of zebrafish after clofibrate treatment.** Staining of peroxisomes in the hepatocytes of zebrafish fed 0% clofibrate **(A)** and 1.00% clofibrate **(B)**. Number of peroxisome per field of view in liver increased with increasing concentration of clofibrate fed zebrafish **(C)**. Arrows point to peroxisomes. Bar = 2 μm.

**Figure 2:**
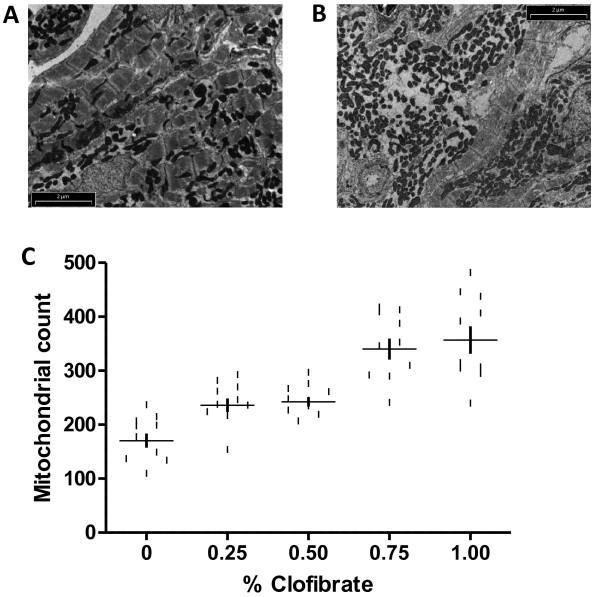
**Electron micrographs of heart cells of zebrafish after clofibrate treatment.** Mitochondria in the heart cells of zebrafish fed 0% clofibrate **(A)** and 1.00% clofibrate **(B)**. Number of mitochondria per field of view in heart increased with increasing concentration of clofibrate fed zebrafish **(C)**. Bar = 2 μm.

Peroxisome proliferators, such as clofibrate, are known to cause a marked proliferation of peroxisomes in the hepatocytes of animals [58-65]. Proliferation of peroxisomes is also associated with a predictable pleiotropic response, characterized by hepatomegaly, and the increased steady-state level of mRNAs coding for peroxisomal enzymes [61]. In this study, we first wished to determine if clofibrate acts as a peroxisome proliferator in zebrafish as vertebrate species show different responses to clofibrate as assayed by peroxisome proliferation or induction of steady-state transcript levels for several clofibrate-responsive genes. Rats and mice are more responsive to clofibrate than hamsters and humans [66,67], while some fish, such as medaka and rainbow trout, show little response [51], and sea bass is essentially refractory to clofibrate treatment [68]. The number of peroxisomes was higher in hepatocytes of zebrafish fed ≥ 0.75% clofibrate (Figure 1B) compared to livers of zebrafish not fed clofibrate (Figure 1A). The number of peroxisomes in liver increased 4-fold in zebrafish fed ≥ 0.75% clofibrate compared to the control (Figure 1C). The peroxisomal numbers in intestine did not change with clofibrate treatment, whereas, in other tissues like muscle, brain and heart, we could not observe any peroxisomes (data not shown). Previous studies in rats and mice fed clofibrate showed an increase in the number of mitochondria in the liver [69-71]. In this study, zebrafish fed ≥ 0.75% clofibrate showed an increase in the number of mitochondria only in heart cells (Figure 2B) compared to the control (Figure 2A). The number of mitochondria in heart cells increased 2-fold in zebrafish fed ≥ 0.75% clofibrate compared to the control (Figure 2C). The mitochondrial number in other tissues (liver, intestine, muscle and brain) examined did not change in zebrafish fed clofibrate.

**Figure 3:**
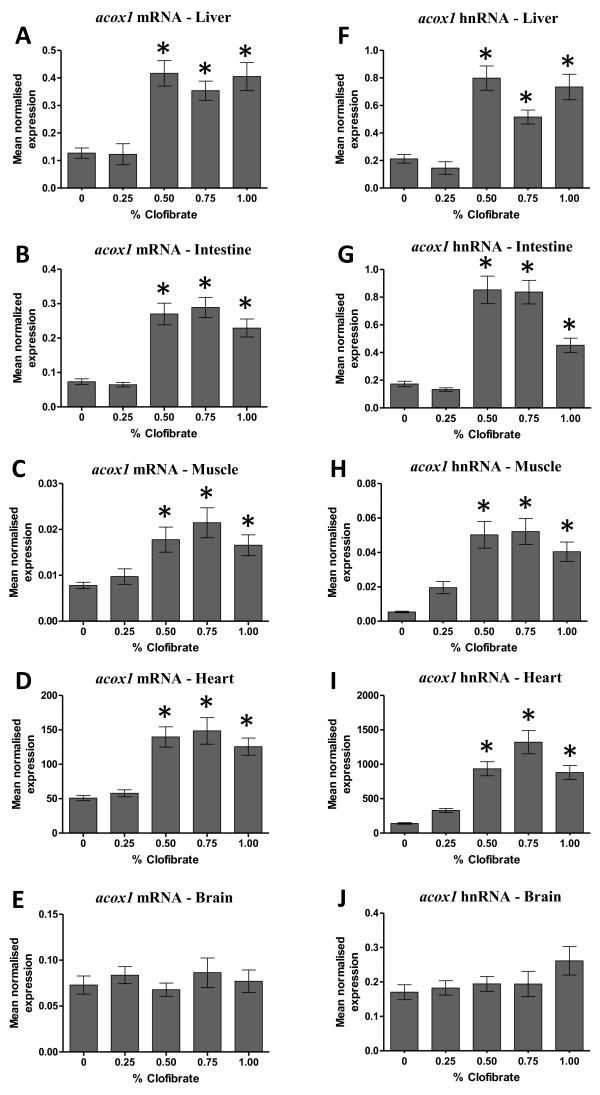
**The steady-state level of * acox1 * mRNA and hnRNA in various tissues of zebrafish fed clofibrate.** The level of mRNA and hnRNA of the *acox1* gene in liver **(A, F)**, intestine **(B, G)**, muscle **(C, H)**, heart **(D, I)** and brain **(E, J)** was determined by RT-qPCR using gene-specific primers. The steady-state level of *acox1* transcripts was normalized to the steady-state level of *rpl13α* transcripts in the same sample. Data are presented as the mean ratio ± S.E.M. Significant differences (*p* < 0.05) in the relative steady-state level of *acox1* mRNA and hnRNA between zebrafish [n = 12, (male = 6, female = 6)] fed different concentrations of clofibrate compared to zebrafish not fed clofibrate are indicated by an asterisk.

Clofibrate has been widely used in vertebrates to activate PPARα, and in some instances PPARγ, to induce transcriptional initiation of genes involved in lipid homeostasis, such as the acyl-CoA oxidase 1 (*Acox1*) gene, a gene that contains a peroxisome proliferator response element (PPRE) [52,65,72,73]. In rats, transcriptional initiation of PPARα-responsive genes are up-regulated in the liver, moderately up-regulated in the small intestine and to a lesser extent up-regulated in other tissues, such as skeletal muscle, heart and kidney by clofibrate [56]. The level of *Acox1* mRNA in liver, heart, kidney, duodenum and jejunum is increased in rats fed clofibrate compared to controls, but not in ileum and brain [56]. Clofibrate was also shown to increase the level of *Acox1* mRNA in liver of chicken [54], liver and adipose tissue of pigs [55], hepatocytes of rainbow trout [63] and liver of rats [64]. In this study, the steady-state level of *acox1* mRNA increased 3-fold in liver (Figure 3A), 3-fold in intestine (Figure 3B), 2-fold in muscle (Figure 3C) and 2.5-fold in heart (Figure 3D) of zebrafish fed ≥ 0.50% clofibrate compared to zebrafish fed < 0.50% clofibrate.

**Figure 4:**
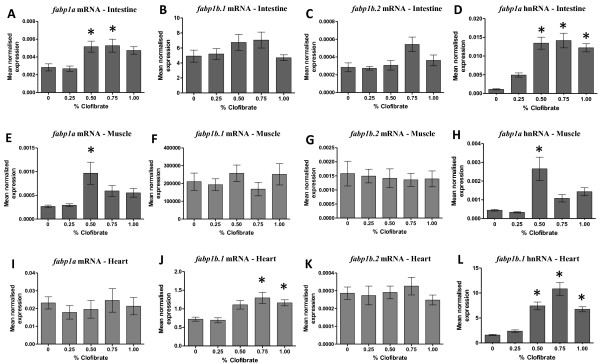
**The steady-state level of * fabp1a/fabp1b.1/fabp1b.2 * mRNA and hnRNA in intestine (A, B, C, D), muscle (E, F, G, H) and heart (I, J, K, L) of zebrafish fed clofibrate.** The level of mRNA and hnRNA was determined by RT-qPCR using gene-specific primers. The steady-state level of *fabp* transcripts was normalized to the steady-state level of *rpl13α* transcripts in the same sample. Data are presented as the mean ratio ± S.E.M. Significant differences (*p* < 0.05) in the relative steady-state level of *fabp* mRNAs between zebrafish [n = 12, (male = 6, female = 6)] fed different concentrations of clofibrate compared to zebrafish not fed clofibrate are indicated by an asterisk.

To determine if the increased levels of *acox1* mRNA transcripts by clofibrate in various tissues was due to an increased rate of transcriptional initiation, we assayed the steady-state level of hnRNA coded by the *acox1* gene. The level of hnRNA for a given gene is an indirect estimate of the rate of transcriptional initiation for that gene as the processing of hnRNA to mRNA occurs rapidly [74]. Zebrafish *acox1* hnRNA increased 2.5-fold in liver (Figure 3F), 2-fold in intestine (Figure 3G), 8-fold in muscle (Figure 3H) and 4-fold in heart (Figure 3I) of fish fed ≥ 0.50% clofibrate compared to zebrafish fed < 0.50% clofibrate indicating an increase of transcriptional initiation of the *acox1* gene in these tissues. In brain, *acox1* mRNA and hnRNA levels did not change in zebrafish fed clofibrate (Figure 3E3J). The lack of affect on the level of *acox1* transcripts in the brain of zebrafish fed clofibrate may be due to: (i) clofibrate does not cross the blood brain barrier, or (ii) if clofibrate does cross the blood brain barrier, the *acox1* gene is not induced by clofibrate in the brain of zebrafish. The increased number of peroxisomes and mitochondria in the liver and heart, respectively, and the induction of the transcriptional initiation of the *acox1* gene by clofibrate in liver, intestine, muscle and heart is compelling evidence that zebrafish is responsive to this peroxisome proliferator, like many other vertebrates [58-72,75].

### Tissue-specific up-regulation of zebrafish *fabp* transcription by clofibrate

Some mammalian *FABP* genes are induced by various FAs and peroxisome proliferators, and molecular mechanisms for their induction have been proposed [46,76-80]. FABPs transport long-chain FAs from the cytoplasm to the nucleus [80,81]. Inside the nucleus, FABPs transfer their long-chain FAs to nuclear receptors, such as PPARα and PPARγ [82-84]. Dietary long chain FAs and peroxisome proliferators activate these nuclear receptors, and once activated, these nuclear receptors form heterodimers with retinoic-acid receptors (RAR) or retinoid X receptors (RXR) (*e.g.,* PPAR-RAR and PPAR-RXR), which in turn bind to response elements in *FABP* genes, and thereby, stimulate initiation of transcription [85-91]. Previous reports have shown that FAs and peroxisome proliferators increase the steady-state level of *L-FABP* (*FABP1*) and *I-FABP* (*FABP2*) gene transcripts in the mammalian liver and small intestine [43,76,77,79,92,93]. Peroxisome proliferators also increase the transcriptional activity of *A-FABP* (*FABP4*) in adipocytes of mice [46,84].

### Up-regulation of transcription of duplicated zebrafish *fabp1* genes by clofibrate

In this study, the steady-state level of *fabp1a* mRNA increased 1.5-fold in the intestine of zebrafish fed 0.50% and 0.75% clofibrate (Figure 4A) and increased 4-fold in muscle of zebrafish fed 0.50% clofibrate compared to zebrafish not fed clofibrate (Figure 4E). In heart cells of zebrafish fed ≥ 0.75% clofibrate, *fabp1b.1* mRNA increased 2-fold compared to zebrafish not fed clofibrate (Figure 4J). To determine if the increased levels of *fabp1a* mRNAs was the result of transcriptional initiation, we assayed the levels of hnRNA for these *fabp* genes in various tissues of zebrafish. The steady-state level of *fabp1a* hnRNA increased 6-fold in intestine of zebrafish fed ≥ 0.50% clofibrate (Figure 4D) and > 5-fold in muscle of zebrafish fed 0.50% clofibrate (Figure 4H). In zebrafish fed ≥ 0.50% clofibrate, the level of *fabp1b.1* hnRNA in heart increased 3-fold compared to zebrafish fed the control diet. (Figure 4L). The levels of *fabp1a* mRNA in heart (Figure 4I), *fabp1b.1* mRNA in intestine (Figure 4B), *fabp1b.1* mRNA in muscle (Figure 4F), *fabp1b.2* mRNA in intestine (Figure 4C), *fabp1b.2* mRNA in muscle (Figure 4G) and *fabp1b.2* mRNA in heart (Figure 4K) remained unchanged in zebrafish fed clofibrate.

### Up-regulation of transcription of zebrafish *fabp7* genes by clofibrate

**Figure 5:**
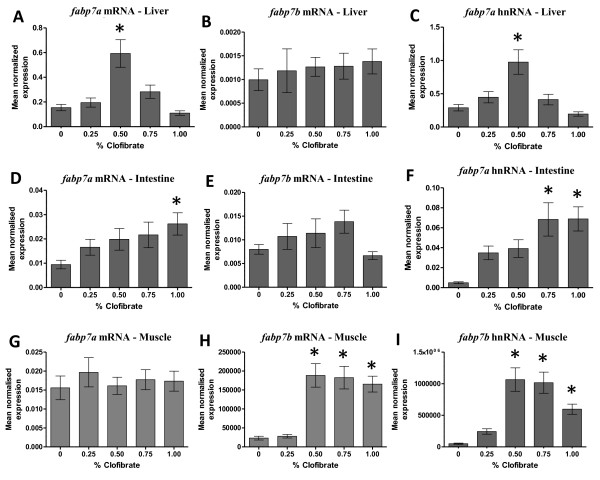
**The steady-state level of * fabp7a/fabp7b * mRNA and hnRNA in liver (A, B, C), intestine (D, E, F) and muscle (G, H, I) of zebrafish fed clofibrate.** The level of mRNA and hnRNA was determined by RT-qPCR using gene-specific primers. The steady-state level of *fabp* transcripts was normalized to the steady-state level of *rpl13α* transcripts in the same sample. Data are presented as the mean ratio ± S.E.M. Significant differences (*p* < 0.05) in the relative steady-state level of *fabp* mRNAs between zebrafish [n = 12, (male = 6, female = 6)] fed different concentrations of clofibrate compared to zebrafish not fed clofibrate are indicated by an asterisk.

Duplicated copies of zebrafish *fabp7* (*fabp7a* and *fabp7b*) exhibited distinct tissue-specific patterns of up-regulation by clofibrate of levels of both mRNA and hnRNA (Figure 5). *fabp7a* mRNA increased > 7-fold in liver of zebrafish fed 0.50% clofibrate (Figure 5A) and > 2-fold in intestine of zebrafish fed 1.00% clofibrate (Figure 5D), while *fabp7b* mRNA levels increased 6-fold only in muscle of zebrafish fed ≥ 0.50% clofibrate compared to zebrafish not fed clofibrate (Figure 5H).

The increase in the mRNA levels of zebrafish *fabp7* genes correlated with the increase in the levels of their hnRNA. *fabp7a* hnRNA increased > 3-fold in liver of zebrafish fed 0.50% clofibrate (Figure 5C) and 7-fold in intestine of zebrafish fed ≥ 0.75% clofibrate (Figure 5F), while *fabp7b* hnRNA increased 6-fold in muscle of zebrafish fed ≥ 0.50% clofibrate (Figure 5I) compared to control zebrafish. No change was observed in the levels of *fabp7b* mRNA transcripts in liver (Figure 5B) and intestine (Figure 5E), and *fabp7a* mRNA in muscle (Figure 5G) in zebrafish fed clofibrate.

### Up-regulation of zebrafish *fabp10* gene transcription by clofibrate

**Figure 6:**
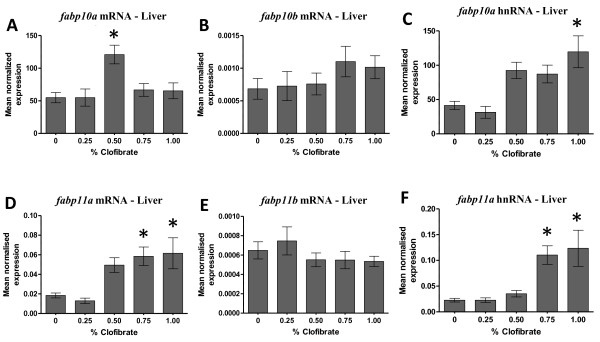
**The steady-state level of * fabp10a/fabp10b * mRNA and hnRNA in liver (A, B, C) and * fabp11a/fabp11b * (mRNA and hnRNA) in liver (D, E, F) of zebrafish fed clofibrate.** The level of mRNA and hnRNA was determined by RT-qPCR using gene-specific primers. The steady-state level of *fabp* transcripts was normalized to the steady-state level of *rpl13α* transcripts in the same sample. Data are presented as the mean ratio ± S.E.M. Significant differences (*p* < 0.05) in the relative steady-state level of *fabp* mRNAs between zebrafish [n = 12, (male = 6, female = 6)] fed different concentrations of clofibrate compared to zebrafish not fed clofibrate are indicated by an asterisk.

The steady-state level of *fabp10a* mRNA increased > 2-fold in liver of zebrafish fed 0.50% clofibrate compared to control (Figure 6A), whereas the level of *fabp10b* mRNA (Figure 6B) did not change in the liver of zebrafish fed clofibrate. A 3-fold increase of *fabp10a* hnRNA mirrored the increase of mRNA coded by this gene in liver of zebrafish fed 1.00% clofibrate compared to control zebrafish (Figure 6C).

### Up-regulation of zebrafish *fabp11* gene transcription by clofibrate

The steady-state level of *fabp11a* mRNA (Figure 6D) increased 3-fold in liver of zebrafish fed ≥ 0.75% clofibrate compared to control, but the steady-state level of *fabp11b* transcripts (Figure 6E) did not change in the liver of zebrafish fed clofibrate. Similarly, *fabp11a* hnRNA increased > 4-fold in liver of zebrafish fed ≥ 0.75% clofibrate compared to control (Figure 6F). No difference in the steady-state level of any *fabp* mRNA and hnRNA assayed was observed between male and female zebrafish (data not shown).

## Conclusion

We report here that zebrafish fed clofibrate exhibited distinct patterns of up-regulation of the steady-state level of mRNAs of duplicated *fabp* genes (Table1). None of the levels of *fabp* mRNA transcripts assayed changed in the brain of zebrafish fed clofibrate (data not shown). Furthermore, changes in the levels of mRNA for a specific *fabp* gene were directly correlated with changes in the steady-state level of hnRNA for that particular *fabp* gene indicating that clofibrate induced transcriptional initiation of zebrafish *fabp* genes (Table1). Clofibrate induction of some zebrafish *fabp* genes appears, however, to be controlled by a tissue-specific mechanism(s), as induction of the steady-state level of *fabp* mRNAs and hnRNAs by clofibrate was seen for both duplicated copies of *fabp1a/fabp1b.1,* and *fabp7a/fabp7b*, but in different tissues. Clofibrate also increased the steady-state level of *fabp10a* and *fabp11a* mRNAs and hnRNAs in liver*,* but not for *fabp10b* and *fabp11b*.

**Table 1:** **Steady-state levels of specific mRNA and hnRNA of *fabp* genes increased in tissues of zebrafish after clofibrate treatment**

**Gene**	**Liver**	**Intestine**	**Muscle**	**Heart**	**Brain**
*acox1*	_**+**_	_**+**_	_**+**_	_**+**_	**-**
*fabp1a*	_** *−* **_	_**+**_	_**+**_	_**−**_	**-**
*fabp1b.1*	_** *−* **_	**-**	**-**	_**+**_	**-**
*fabp1b.2*	_** *−* **_	**-**	**-**	_**−**_	**-**
*fabp7a*	_**+**_	**+**	**-**	_**−**_	**-**
*fabp7b*	_** *−* **_	**-**	_**+**_	_**−**_	**-**
*fabp10a*	_**+**_	**-**	**-**	_**−**_	**-**
*fabp10b*	_** *−* **_	**-**	**-**	_**−**_	**-**
*fabp11a*	_**+**_	**-**	**-**	_**−**_	**-**
*fabp11b*	_** *−* **_	**-**	**-**	_**−**_	**-**

+, increase relative to control.

-, no change relative to control.

Based on *in silico* analyses, we found that most zebrafish *fabp* genes contain putative PPREs within 7 kilobase pairs (kb) of DNA upstream of the transcriptional initiation site of each *fabp* gene (data not shown). Since functional PPREs that activate gene transcription *via* peroxisome proliferators have also been found in introns [73,94], we screened the intronic sequences of each of the zebrafish *fabp* genes described in this paper for PPREs. Many of these *fabp* genes contained putative PPREs in their introns (data not shown). While *in silico* analyses can be very useful in determining the direction of future experimental work, the results of *in silico* analyses must be interpreted cautiously. To illustrate this cautionary point, *in silico* analysis of the rat peroxisomal thiolase B gene identified a putative PPRE in the promoter region of this gene that did not bind PPARα *in vitro*, but subsequent studies showed that a functional PPRE in intron 3 of this gene did bind an activated PPARα *in vitro*[94]. To demonstrate that the putative PPREs we have found by *in silico* analysis in the various zebrafish *fabp* genes are indeed functional will require studies involving deletion of PPREs and/or site directed mutation of putative PPRE sequences in the *fabp* promoters and introns to demonstrate loss of function in various cell culture lines and transgenic zebrafish. If functional PPREs are identified in the zebrafish *fabp* genes, the most parsimonious explanation of the tissue-specific differential induction of transcriptional initiation of the duplicated zebrafish *fabp1a/fabp1b.1* and *fabp7a/fabp7b* genes by clofibrate is that both duplicated copies of these gene have retained a functional PPRE, but that induction by clofibrate is over-ridden by an, as yet, unknown tissue-specific mechanism(s). An alternative explanation is that induction of *fabp* transcriptional initiation by clofibrate is mediated *via* an indirect mechanism wherein the induction of *fabp* genes occurs by an intermediate or “upstream” gene activated by PPAR coding for a transcription factor, which in turn activates zebrafish *fabp* genes. Again, however, this indirect induction of *fabp* gene transcription by clofibrate-activated PPAR must be mediated by an over-riding tissue-specific mechanism(s). Whether clofibrate-induced transcription of zebrafish *fabp* genes is the result of clofibrate-activated PPAR directly at a *fabp* PPRE or indirectly *via* an “upstream” gene coding for a regulatory protein, the regulatory DNA elements in the duplicated *fabp* genes have certainly diverged markedly since the WGD event ~230-400 million years ago [4,13-17], thereby supporting the DDC model [6,7] for the retention of these duplicated *fabp* genes in the zebrafish genome.

## Materials and methods

### Experimental diet and zebrafish husbandry

Experimental diets containing five different concentrations (0, 0.25, 0.50, 0.75 and 1.00% w/w) of clofibrate (Sigma-Aldrich, Oakville, Ontario, Canada) were formulated (Table2). Clofibrate concentrations and basic feed formulation were based on previous dietary studies [57,95-97] and the United States National Research Council’s nutrient requirement recommendations for warm-water fishes [95]. The dry ingredients were mixed using a Hobart mixer for 20 min. Choline chloride was dissolved in distilled water and clofibrate mixed in corn oil prior to addition to dry ingredients. Boiling water was added to the dry ingredients to make wet dough (40% v/v). The dough was spread on tray and freeze-dried for 36–48 h. The freeze-dried diet was then passed through a 0.8 mm mesh to yield particles of less than 800 μm, which were then stored at −20°C.

**Table 2:** Composition of diets (% by weight)

**Ingredients**	**0% clofibrate diet**	**0.25% clofibrate diet**	**0.50% clofibrate diet**	**0.75% clofibrate diet**	**1.00% clofibrate diet**
Vitamin free casein^a^	33	33	33	33	33
Wheat gluten^b^	10	10	10	10	10
Gelatin^a^	4	4	4	4	4
Corn oil^c^	4	4	4	4	4
Fish oil^d^	4	4	4	4	4
Corn starch^e^	33	33	33	33	33
Celufil^1^	8.00	7.75	7.50	7.25	7.00
Vitamin mix^f^	1.30	1.30	1.30	1.30	1.30
Mineral mix^g^	1	1	1	1	1
Betaine^h^	1.50	1.50	1.50	1.50	1.50
DL-Methionine^a^	0.20	0.20	0.20	0.20	0.20
Clofibrate^i^	0	0.25	0.50	0.75	1.00
Total	100	100	100	100	100

^a^ US Biochemical. (Cleveland, OH, USA).

^b^ Dover Mills Ltd. (Halifax, NS, Canada).

^c^ Sobeys Inc. (Halifax, NS, Canada).

^d^ Corey Feeds Ltd. (Fredericton, NB, Canada).

^e^ National Starch and Chemical Co. (Bridgewater, NJ, USA).

^f^ Vitamin added to supply the following (per kg diet): vitamin A, 8000 IU; vitamin D3, 4000 IU; vitamin E, 300 IU; menadione sodium bisulfite, 40 mg; Thiamine HCl, 50 mg; riboflavin, 70 mg; d-Ca pantothenate, 200 mg; biotin, 1.5 mg; folic acid, 20 mg; vitamin B12, 0.15 mg; niacin, 300 mg; pyridoxine HCl, 20 mg; ascorbic acid, 300 mg; inositol, 400 mg; choline chloride, 2000 mg; butylated hydroxy toluene, 15 mg; butylated hydroxy anisole, 15 mg.

^g^ Mineral added to supply the following (per kg diet): manganous sulphate (32.5% Mn), 40 mg; ferrous sulphate (20.1% Fe), 30 mg; copper sulphate (25.4% Cu), 5 mg; zinc sulphate (22.7% Zn), 75 mg; sodium selenite (45.6% Se), 1 mg; cobalt chloride (24.8% Co), 2.5 mg; sodiumfluoride (42.5% F), 4 mg.

^h^ Betaine anhydrous (96% feed grade). (Finnfeeds, Finland). ^i^ Sigma-Aldrich Inc. (St. Louis, MO, USA).

To reduce genetic variance, four female and two male adult zebrafish of the AB strain [98], obtained from the Aquatron at Dalhousie University, were bred in a single tank to produce embryos. Embryos, larvae and adult fish were maintained in aerated water at 28.5°C on a 14 h light and 10 h dark cycle [99]. One hundred and fifty day-old zebrafish were acclimatized in 35 L aquaria for four weeks prior to feeding fish diets containing clofibrate. Three replicates of five different dietary groups of fish were distributed in 15 tanks in a randomized complete block design. Each tank contained 15 zebrafish. Fish in each tank were maintained under the same light intensity and photoperiod. After acclimatization for a week, fish were fed the experimental diets twice a day to satiation. At the end of four weeks, fish were anaesthetized by immersion in a solution of 0.20% (v/v) MS-222 prior to tissue dissection. Dissection of fish was done on ice. From each fish, liver, intestine, muscle, brain and heart were removed. All experimental protocols were approved by the Dalhousie University Committee on Laboratory Animals in accordance with the recommendations of the Canadian Council on Animal Care.

### Electron microscopy to visualize peroxisomes and mitochondria

Tissue samples (liver, intestine, muscle, brain and heart) from 0, 0.25, 0.50, 0.75 and 1.00% (w/w) clofibrate-fed fish were dissected and transferred to centrifuge tubes containing 2% glutraldehyde fixative (osmolarity ~300 mOsm) for 30–40 min on ice [100]. The tissue samples were subjected to three 10-minute washes in 0.1 M cacodylate buffer. The samples were transferred to 0.1 M Tris–HCl buffer and washed twice for 10 min. The samples were pre-incubated in 1% diaminobenzidine (DAB) solution for 30 min at 37°C with shaking. Ten μl of 30% hydrogen peroxide solution was added and the samples were incubated for 20–30 min at 37°C with shaking. Tissues were washed in 0.1 M TBS for 10 min and transferred to centrifuge tubes and subjected to three 10-minute washes with 0.1 M cacodylate buffer at room temperature [101]. Finally, the tissues were post-fixed in 1% osmium in 0.1 M cacodylate buffer for 1 h at 4°C and washed in filtered, deionized H_2_O for 15 min. Tissues were dehydrated for transmission electron microscopy, infiltrated overnight and later embedded in epon resin [102]. Ten electron microscopy images of tissues for peroxisomes and mitochondria were counted in tissues of adult zebrafish fed different concentrations of clofibrate.

### RNA isolation, cDNA synthesis and RT-qPCR

Total RNA was extracted from adult zebrafish tissues using TRIzol (Invitrogen, Carlsbad, CA, USA) according to the protocol recommended by the supplier. The quality and quantity of extracted RNA was assessed by agarose gel-electrophoresis and spectrophotometry at 260 nm, respectively. cDNA was synthesized from mRNA using an oligo (dT) primer according to the manufacturer’s protocol for the omniscript RT kit (Qiagen, Mississauga, Canada). cDNA was synthesized from hnRNA using random hexamers. Primer sequences for the quantification of mRNA and hnRNA encoded by different *fabp* genes and their annealing temperature (AT) for primer pairs of each *fabp* gene during PCR are shown in Table3. To assay specific hnRNAs, one primer was complementary to an intronic sequence, while the other was complementary to an exonic sequence. *acox1*, a gene known to be induced by clofibrate in many organisms [52,72] was used as a positive control.

**Table 3:** Primer sequences used for RT-qPCR

**Gene symbol**	**Entrez Gene ID**	**Forward primer 5' -› 3'**	**Reverse primer 5' -› 3'**	**AT**^**a**^
mRNA quantification				
*fabp1a*	791610	TAAGCTGACAGCGTTTGTGAAGGG	AGATGCGTCTGCTGATCCTCTTGT	60.0
*fabp1b.1*	554095	AAGCTGAAGGTGGTGCTGAACA	CACGTTTGCTGATGCGCTTGTA	59.0
*fabp1b.2*	EB880179	TGCCGTTCTCTGGGAAGTTTGAGT	TGACTTTGTCTCCGCTCAGCATCT	61.0
*fabp7a*	58128	TGTGCCACTTGGAAACTGGTTGAC	AACATTGCCTACTTGCCTGGTAGG	60.0
*fabp7b*	407736	AAACCACTGCTGATGACCGACACT	AGTGGTCTCTTTCCCATCCCACTT	61.0
*fabp10a*	171481	TTACGCTCAGGAGAACTACG	CTTCCTGATCATGGTGGTTC	55.0
*fabp10b*	795210	CGGCTCCAGAGCACTACATC	GTTCACTCATGTGCGGGAGC	60.0
*fabp11a*	447944	TGTGCAGAAACAGACCTGGGA	ACAGCCACCACATCACCCATCTT	60.0
*fabp11b*	553579	GCTGTCACTACATTCAAGACCTG	AGTTTACCATCCGCAAGGCTCA	60.0
*acox1*	449662	AGTCAGCACGAGCTCTCTCC	GCCCTACAAAGTGAAAGGCA	58.0
*rpl13α*	560828	AGCAAGTGCTGTTGGGCCAC	GTGTGGCGGTGATGGCCTGG	61.0
hnRNA quantification				
*fabp1a*	791610	ATCAATGGAGGTCAACGGCGAC	CAGCATGCGTGAAGCCGCCC	62.5
*fabp1b.1*	554095	GAACTAACGTGTGCTGCTTGTG	CACGTTTGCTGATGCGCTTGTA	57.0
*fabp7a*	58128	CCATCCATCAGATTTCTATGTGGG	CATTATGCCTTCTCGTATGTGCG	56.5
*fabp7b*	407736	TTGGAAATGTGACCAAACCGACGC	TCGTCTCGAAAGGGAATGCAGTGT	61.5
*fabp10a*	171481	TCCAGCAGAACGGCAGCGAC	CGCCTGTAAAGTGAAGCCATTTCCA	61.0
*fabp11a*	447944	CCAAGCCGTTTTTGATGATGTGAG	GCTATTAATTTCCCATCCGACACC	57.0
*acox1*	449662	GGCTACTCCCGCTGCAGCAG	GGCCTGAGGGTTGTTGGGCC	63.0
*rpl13α*	560828	ACCAACCCTTCCCGTGGACCA	AGCCAATGCTTGCTTCTACAACAGA	61.5

^a^AT, annealing temperature (°C).

Amplification of cDNA samples and DNA standards was carried out using the QuantiTect SYBR Green PCR Kit (Qiagen, Mississauga, Ontario, Canada) following the manufacturer's instructions. For thermal cycling and fluorescence detection, a Rotor-Gene 3000 system (Corbett Research, Sydney, Australia) was used. PCR conditions were: initial hold for 15 min at 95°C followed by 40 cycles of 15 s denaturation at 94°C, 20 s annealing of primers at different temperatures depending on the primer pairs (see Table3), and 30 s of elongation at 72°C. Following completion of the PCR cycles, the melting temperature of the PCR product was determined as an indication that total fluorescence was derived from a single gene-specific product. Fluorescence was measured following each cycle. The copy number of mRNA and hnRNA for each *fabp* gene was determined using the standard curves as explained by Bustin *et al.*[103]. As negative controls, reverse transcriptase was omitted from cDNA synthesis reactions for each sample and these controls were subjected to quantitative PCR. To determine the relative steady-state level of *fabp* mRNA and hnRNA transcripts in each tissue, the absolute copy number of *fabp* mRNA and hnRNA transcripts was divided by the copy number of ribosomal protein large subunit 13α (*rpl13α*) [104] mRNA and hnRNA transcripts in each sample.

### Statistical analysis

Statistical analyses were performed using the GraphPad PRISM® software version-5 (San Diego, California, USA). Data were analyzed using one-way analysis of variance (ANOVA). *Post hoc* comparisons were conducted using the Tukey’s Multiple Comparison Test. The level of significance was chosen at *p* < 0.05 and the results were presented as means ± S.E.M.

## Abbreviations

DDC: Duplication-degeneration-complementation; WGD: Whole genome duplication; FAs: Fatty acids; hnRNA: Heterogenous nuclear RNA; FABP: Mammal fatty acid-binding protein; FABP: Mammal fatty acid-binding protein gene; Fabp: Fish fatty acid-binding protein; Fabp: Fish fatty acid-binding protein gene; PPAR: Peroxisome proliferator-activated receptor; PPRE: Peroxisome proliferator response element; RT-qPCR: Reverse transcription-quantitative polymerase chain reaction.

## Competing interest

The authors declare that they have no competing interests.

## Author’s contributions

ABV and JMW conceived and designed the studies. ABV carried out the experimental work and statistical analysis. SPL assisted in the formulation of zebrafish diet. EMD-W assisted in design and interpretation of RT-qPCR analysis. ABV and JMW drafted the manuscript. All authors read and approved the final version of the manuscript.

## References

[B1] Ohno S Evolution by Gene Duplication 1970 New York (NY): Springer

[B2] Holland PW Garcia-Fernandez J Williams NA Sidow A Gene duplications and the origins of vertebrate development Dev Suppl 1994 125 133 7579513

[B3] Sidow A Gen(om)e duplications in the evolution of early vertebrates Curr Opin Genet Dev 1996 6 715 722 10.1016/S0959-437X(96)80026-8 8994842

[B4] Robinson-Rechavi M Marchand O Escriva H Bardet PL Zelus D Hughes S Laudet V Euteleost fish genomes are characterized by expansion of gene families Genome Res 2001 11 781 788 10.1101/gr.165601 11337474 PMC311060

[B5] Taylor JS Raes J Duplication and divergence: the evolution of new genes and old ideas Annu Rev Genet 2004 38 615 643 10.1146/annurev.genet.38.072902.092831 15568988

[B6] Force A Lynch M Pickett FB Amores A Yan YL Postlethwait J Preservation of duplicate genes by complementary, degenerative mutations Genetics 1999 151 1531 1545 10101175 10.1093/genetics/151.4.1531 PMC1460548

[B7] Lynch M Conery JS The evolutionary fate and consequences of duplicate genes Science 2000 290 1151 1155 10.1126/science.290.5494.1151 11073452

[B8] Bernlohr DA Simpson MA Hertzel AV Banaszak LJ Intracellular lipid-binding proteins and their genes Annu Rev Nutr 1997 17 277 303 10.1146/annurev.nutr.17.1.277 9240929

[B9] Schaap FG van der Vusse GJ Glatz JFC Evolution of the family of intracellular lipid-binding proteins in vertebrates Mol Cell Biochem 2002 239 69 77 10.1023/A:1020519011939 12479570

[B10] Haunerland NH Spener F Fatty acid-binding proteins - insights from genetic manipulations Prog Lipid Res 2004 43 328 349 10.1016/j.plipres.2004.05.001 15234551

[B11] Wolfrum C Cytoplasmic fatty acid-binding protein sensing fatty acids for peroxisome proliferator activated receptor activation Cell Mol Life Sci 2007 64 2465 2467 10.1007/s00018-007-7279-4 17876520 PMC11136282

[B12] Storch J Corsico B The emerging functions and mechanisms of mammalian fatty acid-binding proteins Annu Rev Nutr 2008 28 73 95 10.1146/annurev.nutr.27.061406.093710 18435590

[B13] Furlong RF Holland PWH Were vertebrates octoploid? Philos Trans R Soc Lond B Biol Sci 2002 357 531 544 10.1098/rstb.2001.1035 12028790 PMC1692965

[B14] Jaillon O Aury JM Brunet F Petit JL Stange-Thomann N Mauceli E Bouneau L Fischer C Ozouf-Costaz C Bernot A Nicaud S Jaffe D Fisher S Lutfalla G Dossat C Segurens B Dasilva C Salanoubat M Levy M Boudet N Castellano S Anthouard V Jubin C Castelli V Katinka M Vacherie B Biémont C Skalli Z Cattolico L Poulain J de Berardinis V Cruaud C Duprat S Brottier P Coutanceau JP Gouzy J Parra G Lardier G Chapple C McKernan KJ McEwan P Bosak S Kellis M Volff JN Guigó R Zody MC Mesirov J Lindblad-Toh K Birren B Nusbaum C Kahn D Robinson-Rechavi M Laudet V Schachter V Quétier F Saurin W Scarpelli C Wincker P Lander ES Weissenbach J Roest Crollius H Genome duplication in the teleost fish *Tetraodon nigroviridis* reveals the early vertebrate protokaryotype Nature 2004 431 946 957 10.1038/nature03025 15496914

[B15] Woods IG Kelly PD Chu F Ngo-Hazelett P Yan Y-L Huang H Postlethwait JH Talbot WS A comparative map of the zebrafish genome Genome Res 2000 10 1903 1914 10.1101/gr.10.12.1903 11116086 PMC313070

[B16] Christoffels A Koh EG Chia JM Brenner S Aparicio S Venkatesh B Fugu genome analysis provides evidence for a whole-genome duplication early during the evolution of ray-finned fishes Mol Biol Evol 2004 21 1146 1151 10.1093/molbev/msh114 15014147

[B17] Vandepoele K De Vos W Taylor JS Meyer A Van de Peer Y Major events in the genome evolution of vertebrates: Paranome age and size differ considerably between ray-finned fishes and land vertebrates PNAS 2004 101 1638 1643 10.1073/pnas.0307968100 14757817 PMC341801

[B18] Denovan-Wright EM Pierce M Wright JM Nucleotide sequence of cDNA clones coding for a brain-type fatty acid-binding protein and its tissue-specific expression in adult zebrafish (*Danio rerio*) Biochim Biophys Acta - Gene Struct Expr 2000 1492 221 226 10.1016/S0167-4781(00)00075-0 11004493

[B19] Denovan-Wright EM Pierce M Sharma MK Wright JM cDNA sequence and tissue-specific expression of a basic liver-type fatty acid-binding protein in adult zebrafish (*Danio rerio*) Biochim Biophys Acta - Gene Struct Expr 2000 1492 227 232 10.1016/S0167-4781(00)00102-0 11004494

[B20] Pierce M Wang YM Denovan-Wright EM Wright JM Nucleotide sequence of a cDNA clone coding for an intestinal-type fatty acid binding protein and its tissue-specific expression in zebrafish (*Danio rerio*) Biochim Biophys Acta - Gene Struct Expr 2000 1490 175 183 10.1016/S0167-4781(99)00229-8 10786634

[B21] Liu RZ Denovan-Wright EM Wright JM Structure, mRNA expression and linkage mapping of the brain-type fatty acid-binding protein gene (*fabp7*) from zebrafish (*Danio rerio*) Eur J Biochem 2003 270 715 725 10.1046/j.1432-1033.2003.03432.x 12581211

[B22] Liu RZ Denovan-Wright EM Wright JM Structure, linkage mapping and expression of the heart-type fatty acid-binding protein gene (*fabp3*) from zebrafish (*Danio rerio*) Eur J Biochem 2003 270 3223 3234 10.1046/j.1432-1033.2003.03705.x 12869198

[B23] Liu RZ Denovan-Wright EM Degrave A Thisse C Thisse B Wright JM Differential expression of duplicated genes for brain-type fatty acid-binding proteins (*fabp7a and fabp7b*) during early development of the CNS in zebrafish (*Danio rerio*) Gene Expr Patterns 2004 4 379 387 10.1016/j.modgep.2004.01.010 15183304

[B24] Sharma MK Denovan-Wright EM Degrave A Thisse C Thisse B Wright JM Sequence, linkage mapping and early developmental expression of the intestinal-type fatty acid-binding protein gene (*fabp2*) from zebrafish (*Danio rerio*) Comp Biochem Physiol B Biochem Mol Biol 2004 138 391 398 10.1016/j.cbpc.2004.05.009 15325340

[B25] Sharma MK Liu RZ Thisse C Thisse B Denovan-Wright EM Wright JM Hierarchical subfunctionalization of *fabp1a, fabp1b* and *fabp10* tissue-specific expression may account for retention of these duplicated genes in the zebrafish (*Danio rerio*) genome FEBS J 2006 273 3216 3229 10.1111/j.1742-4658.2006.05330.x 16857010

[B26] Liu RZ Saxena V Sharma MK Thisse C Thisse B Denovan-Wright EM Wright JM The *fabp4* gene of zebrafish (*Danio rerio*) - genomic homology with the mammalian *FABP4* and divergence from the zebrafish *fabp3* in developmental expression FEBS J 2007 274 1621 1633 10.1111/j.1742-4658.2007.05711.x 17480210

[B27] Alves-Costa FA Denovan-Wright EM Thisse C Thisse B Wright JM Spatio-temporal distribution of fatty acid-binding protein 6 (*fabp6*) gene transcripts in the developing and adult zebrafish (*Danio rerio*) FEBS J 2008 275 3325 3334 10.1111/j.1742-4658.2008.06480.x 18492067

[B28] Karanth S Denovan-Wright EM Thisse C Thisse B Wright JM The evolutionary relationship between the duplicated copies of the zebrafish fabp11 gene and the tetrapod *FABP4*, *FABP5*, *FABP8* and *FABP9* genes FEBS J 2008 275 3031 3040 10.1111/j.1742-4658.2008.06455.x 18445037

[B29] Venkatachalam AB Thisse C Thisse B Wright JM Differential tissue-specific distribution of transcripts for the duplicated fatty acid-binding protein 10 (*fabp10*) genes in embryos, larvae and adult zebrafish (*Danio rerio*) FEBS J 2009 276 6787 6797 10.1111/j.1742-4658.2009.07393.x 19843178

[B30] Karanth S Denovan-Wright EM Thisse C Thisse B Wright JM Tandem duplication of the fabp1b gene and subsequent divergence of the tissue-specific distribution of *fabp1b.1* and *fabp1b.2* transcripts in zebrafish (Danio rerio) Genome 2009 52 985 992 10.1139/G09-071 19953126

[B31] Postlethwait JH Woods IG Ngo-Hazelett P Yan YL Kelly PD Chu F Huang H Hill-Force A Talbot WS Zebrafish comparative genomics and the origins of vertebrate chromosomes Genome Res 2000 10 1890 1902 10.1101/gr.164800 11116085

[B32] Woods IG Wilson C Friedlander B Chang P Reyes DK Nix R Kelly PD Chu F Postlethwait JH Talbot WS The zebrafish gene map defines ancestral vertebrate chromosomes Genome Res 2005 15 1307 1314 10.1101/gr.4134305 16109975 PMC1199546

[B33] Hertzel AV Bernlohr DA The mammalian fatty acid-binding protein multigene family: molecular and genetic insights into function Trends Endocrinol Metab 2000 11 175 180 10.1016/S1043-2760(00)00257-5 10856918

[B34] Glatz JF van der Vusse GJ Cellular fatty acid-binding proteins: their function and physiological significance Prog Lipid Res 1996 35 243 282 10.1016/S0163-7827(96)00006-9 9082452

[B35] Ong DE Newcomer ME Chytil F Sporn MB, Roberts AB, Goodman DS The Retinoids: Biology, Chemistry and Medicine Cellular retinoid-binding proteins 1994 2 Raven, New York 283 317

[B36] Wu Q Andolfatto P Haunerland NH Cloning and sequence of the gene encoding the muscle fatty acid binding protein from the desert locust, *Schistocerca gregaria* Insect Biochem Mol Biol 2001 31 553 562 10.1016/S0965-1748(00)00158-2 11267894

[B37] Parmar MB Venkatachalam AB Wright JM The evolutionary relationship of the transcriptionally-active fabp11a (intron-less) and *fabp11b genes* of medaka with *fabp11* genes of other teleost fishes FEBS J 2012 in press 10.1111/j.1742-4658.2012.08611.x 22520026

[B38] Ong DE Cellular transport and metabolism of vitamin A: roles of the cellular retinoid-binding proteins Nutr Rev 1994 52 S24 31 8202279 10.1111/j.1753-4887.1994.tb01383.x

[B39] Veerkamp JH Maatman RG Cytoplasmic fatty acid-binding proteins: their structure and genes Prog Lipid Res 1995 34 17 52 10.1016/0163-7827(94)00005-7 7644552

[B40] Zimmerman AW Veerkamp JH New insights into the structure and function of fatty acid binding proteins Cell Mol Life Sci 2002 11 1096 1116 12222958 10.1007/s00018-002-8490-y PMC11337517

[B41] Leaver MJ Boukouvala E Antonopoulou E Diez A Favre-Krey L Ezaz MT Bautista JM Tocher DR Krey G Three peroxisome proliferators activated receptor isotypes from each of two species of marine fish Endocrinology 2005 146 3150 3162 10.1210/en.2004-1638 15790725

[B42] Meunier-Durmort C Poirier H Niot I Forest C Besnard P Up-regulation of the expression of the gene for liver fatty acid-binding protein by long-chain fatty acids Biochem J 1996 319 483 487 8912685 10.1042/bj3190483 PMC1217794

[B43] Poirier H Niot I Monnot MC Braissant O Meunier-Durmort C Costet P Pineau T Wahli W Willson TM Besnard P Differential involvement of peroxisome proliferator-activated receptors α and δ in fibrate and fatty-acid-mediated inductions of the gene encoding liver fatty acid-binding protein in the liver and the small intestine Biochem J 2001 355 481 488 10.1042/0264-6021:3550481 11284737 PMC1221761

[B44] Wu Q Haunerland NH A novel fatty acid response element controls the expression of the flight muscle FABP gene of the desert locust, *Schistocerca gregaria* Eur J Biochem 2001 268 5894 5900 10.1046/j.0014-2956.2001.02538.x 11722577

[B45] Qu H Cui L Haunerland JR Haunerland NH Fatty acid-dependent expression of the muscle FABP gene - comparative analysis of gene control in functionally related, but evolutionary distant animal systems Mol Cell Biochem 2007 299 45 53 10.1007/s11010-005-9036-z 17001452

[B46] Schachtrup C Emmler T Bleck B Sandqvist A Spener F Functional analysis of peroxisome-proliferator-responsive element motifs ingenes of fatty acid-binding proteins Biochem J 2004 382 239 245 10.1042/BJ20031340 15130092 PMC1133936

[B47] Her GM Yeh YH Wu JL 435-bp liver regulatory sequence in the liver fatty acid binding protein (L-FABP) gene is sufficient to modulate liver regional expression in transgenic zebrafish Dev Dyn 2003 227 347 356 10.1002/dvdy.10324 12815620

[B48] Her GM Yeh YH Wu JL Functional conserved elements mediate intestinal-type fatty acid binding protein (I-FABP) expression in the gut epithelia of zebrafish larvae Dev Dyn 2004 230 734 742 10.1002/dvdy.20081 15254907

[B49] Karanth S Lall SP Denovan-Wright EM Wright JM Differential transcriptional modulation of duplicated fatty acid-binding protein genes by dietary fatty acids in zebrafish (*Danio rerio*): evidence for subfunctionalization and neofunctionalization of duplicated genes BMC Evol Biol 2009 9 219 10.1186/1471-2148-9-219 19725974 PMC2754478

[B50] Yamoto T Ohashi Y Furukawa T Teranishi M Manabe S Makita T Change of the sex-dependent response to clofibrate in F344 rat liver during postnatal development Toxicol Lett 1996 85 77 83 10.1016/0378-4274(96)03643-0 8650696

[B51] Haasch ML Henderson MC Buhler DR Induction of lauric acid hydroxylase activity in catfish and bluegill by peroxisome proliferating agents Comp Biochem Physiol C Pharmacol Toxicol Endocrinol 1998 121 297 303 10.1016/S0742-8413(98)10050-6 9972471

[B52] Akbiyik F Cinar K Demirpence E Ozsullu T Tunca R Haziroglu R Yurdaydin C Uzunalimoglu O Bozkaya H Ligand-induced expression of peroxisome proliferator-activated receptor alpha and activation of fatty acid oxidation enzymes in fatty liver Eur J Clin Invest 2004 34 429 435 10.1111/j.1365-2362.2004.01359.x 15200495

[B53] Nunes B Carvalho F Guilhermino L Acute and chronic effects of clofibrate and clofibric acid on the enzymes acetylcholinesterase, lactate dehydrogenase and catalase of the mosquitofish, *Gambusia holbrooki* Chemosphere 2004 57 1581 1589 10.1016/j.chemosphere.2004.09.018 15519403

[B54] Konig B Kluge H Haase K Brandsch C Stangl GI Eder K Effects of clofibrate treatment in laying hens Poult Sci 2007 86 1187 1195 17495091 10.1093/ps/86.6.1187

[B55] Luci S Giemsa B Kluge H Eder K Clofibrate causes an upregulation of PPAR-α target genes but does not alter expression of SREBP target genes in liver and adipose tissue of pigs Am J Physiol Regul Integr Comp Physiol 2007 293 R70 R77 10.1152/ajpregu.00603.2006 17363680

[B56] Ringseis R Posel S Hirche F Eder K Treatment with pharmacological peroxisome proliferator-activated receptor α agonist clofibrate causes upregulation of organic cation transporter 2 in liver and small intestine of rats Pharmacol Res 2007 56 175 183 10.1016/j.phrs.2007.06.001 17644405

[B57] Rorvik KA Alne H Gaarder M Ruyter B Maseide NP Jakobsen JV Berge RK Sigholt T Thomassen MS Does the capacity for energy utilization affect the survival of post-smolt atlantic salmon, *Salmo salar L*., during natural outbreaks of infectious pancreatic necrosis J Fish Dis 2007 30 399 409 10.1111/j.1365-2761.2007.00823.x 17584437

[B58] Hess R Staubli W Riess W Nature of the hepatomegalic effect produced by ethyl-chlorophenoxy-isobutylate in the rat Nature 1965 208 856 858 10.1038/208856a0 5870099

[B59] Svoboda DJ Azarnoff DL Response of hepatic microbodies to a hypolipidemic agent, ethyl chlorophenoxy isobutyrate (CPIB) J Cell Biol 1966 30 442 450 10.1083/jcb.30.2.442 5968981 PMC2107000

[B60] Lazarow PB de Duve C A fatty acyl-CoA oxidizing system in rat liver peroxisomes; enhancement by clofibrate, a hypolipidemic drug Proc Natl Acad Sci USA 1976 73 2043 2046 10.1073/pnas.73.6.2043 180535 PMC430444

[B61] Alvares K Carrillo A Yuan PM Kawano H Morimoto RI Reddy JK Identification of cytosolic peroxisome proliferator binding protein as a member of the heat shock protein HSP70 family Proc Natl Acad Sci USA 1990 87 5293 5297 10.1073/pnas.87.14.5293 2371272 PMC54309

[B62] Tanaka K Smith PF Stromberg PC Eydelloth RS Herold EG Grossman SJ Frank JD Hertzog PR Soper KA Keenan KP Studies of early hepatocellular proliferation and peroxisomal proliferation in Sprague–Dawley rats treated with tumorigenic doses of clofibrate Toxicol Appl Pharmacol 1992 116 71 77 10.1016/0041-008X(92)90146-J 1529455

[B63] Donohue M Baldwin LA Leonard DA Kostecki PT Calabrese J Effect of hypolipidemic drugs gemfibrozil, ciprofibrate, and clofibric acid on peroxisomal beta-oxidation in primary cultures of rainbow trout hepatocytes Ecotoxicol Environ Saf 1993 26 127 132 10.1006/eesa.1993.1044 7504609

[B64] Paul HS Sekas G Winters SJ Role of testosterone in the induction of hepatic peroxisome proliferation by clofibrate Metabolism 1994 43 168 173 10.1016/0026-0495(94)90240-2 8121297

[B65] Ibabe A Herrero A Cajaraville MP Modulation of peroxisome proliferator-activated receptors (PPARs) by PPARα- and PPARγ- specific ligands and by 17β-estradiol in isolated zebrafish hepatocytes Toxicol In Vitro 2005 19 725 735 10.1016/j.tiv.2005.03.019 15964169

[B66] Mizumoto K Kitazawa S Eguchi T Nakajima A Tsutsumi M Ito S Danda A Konishi Y Modulation of N-nitrosobis (2-hydroxypropyl) amine-induced carcinogenesis by clofibrate in hamsters Carcinogenesis 1988 9 1421 1425 10.1093/carcin/9.8.1421 3402039

[B67] Holden PR Tugwood JD Peroxisome proliferator-activated receptor alpha: role in rodent liver cancer and species differences J Mol Endocrinol 1999 22 1 8 10.1677/jme.0.0220001 9924174

[B68] Pretti C Novi S Longo V Gervasi PG Effect of clofibrate, a peroxisome proliferator, in sea bass (*Dicentrarchus labrax*), a marine fish Environ Res 1999 80 294 296 10.1006/enrs.1998.3893 10092449

[B69] Lundgren B Bergstrand A Karlsson K DePierre JW Effects of dietary treatment with clofibrate, nafenopin or WY-14.643 on mitochondria and DNA in mouse liver Biochim Biophys Acta 1990 1035 132 138 10.1016/0304-4165(90)90107-8 2393663

[B70] Meijer J Starkerud C Grandell I Afzelius BA Time-dependent effects of the hypolipidemic agentclofibrate on peroxisomes and mitochondria in mouse hepatocytes J Submicroscop Cytol Pathol 1991 23 185 194 2070346

[B71] Eagles DA Chapman GB A light- and electron-microscope study of hepatocytes of rats fed different diets C R Biol 2007 330 62 70 10.1016/j.crvi.2006.09.004 17241949

[B72] Reddy JK Hashimoto T Peroxisomal β-oxidation and peroxisome proliferator-activated receptor α: an adaptive metabolic system Annu Rev Nutr 2001 21 193 230 10.1146/annurev.nutr.21.1.193 11375435

[B73] Helledie T Grøntved L Jensen SS Kiilerich P Rietveld L Albrektsen T Boysen MS Nøhr J Larsen LK Fleckner J Stunnenberg HG Kristiansen K Mandrup S The gene encoding the Acyl-CoA-binding protein is activated by peroxisome proliferator-activated receptor gamma through an intronic response element functionally conserved between humans and rodents J Biol Chem 2002 277 26821 26830 10.1074/jbc.M111295200 12015306

[B74] Watson JD Baker TA Bell SP Gann A Levine M Losick R Molecular Biology of the Gene 2008 Cold Spring Harbor, NY: Pearson Benjamin Cummings/Cold Spring Harbor Laboratory Press 415 421

[B75] Colton HM Falls JG Ni H Kwanyuen P Creech D McNeil E Casey WM Hamilton G Cariello NF Visualization and quantitation of peroxisomes using fluorescent nanocrystals: treatment of rats and monkeys with fibrates and detection in the liver Toxicol Sci 2004 80 183 192 10.1093/toxsci/kfh144 15084755

[B76] Ockner RK Manning JA Fatty acid-binding protein in small intestine. Identification, isolation, and evidence for its role in cellular fatty acid transport J Clin Invest 1974 54 326 338 10.1172/JCI107768 4211161 PMC301560

[B77] Bass NM Manning JA Ockner RK Gordon JI Seetharam S Alpers DH Regulation of the biosynthesis of 2 distinct fatty acid-binding proteins in rat-liver and intestine - influences of sex difference and of clofibrate J Biol Chem 1985 260 1432 1436 3968078

[B78] Reddy JK Peroxisome proliferators and peroxisome proliferator-activated receptor alpha: biotic and xenobiotic sensing Am J Pathol 2004 164 2305 2321 10.1016/S0002-9440(10)63787-X 15161663 PMC1615758

[B79] Mochizuki K Mochizuki H Kawai H Ogura Y Shmada M Takase S Goda T Possible role of fatty acids in milk as the regulator of the expression of cytosolic binding proteins for fatty acids and vitamin A through PPAR alpha in developing rats J Nutr Sci Vitaminol 2007 53 515 521 10.3177/jnsv.53.515 18202540

[B80] Schroeder F Petrescu AD Huang H Atshaves BP McIntosh AL Martin GG Hostetler HA Vespa A Landrock D Landrock KK Role of fatty acid binding proteins and long chain fatty acids in modulating nuclear receptors and gene transcription Lipids 2008 43 1 17 10.1007/s11745-007-3111-z 17882463

[B81] Huang H Starodub O McIntosh A Atshaves BP Woldegiorgis G Kier AB Schroeder F Liver fatty acid-binding protein colocalizes with peroxisome proliferator activated receptor alpha and enhances ligand distribution to nuclei of living cells Biochemistry 2004 43 2484 2500 10.1021/bi0352318 14992586

[B82] Delva L Bastie JN Rochette-Egly C Kraiba R Balitrand N Despouy G Chambon P Chomienne C Physical and functional interactions between cellular retinoic acid binding protein II and the retinoic acid-dependent nuclear complex Mol Cell Biochem 1999 19 7158 7167 10.1128/mcb.19.10.7158 PMC84709 10490651

[B83] Budhu AS Noy N Direct channelling of retinoic acid between cellular retinoic acid binding protein II and retinoic acid receptor sensitizes mammary carcinoma cells to retinoic acid induced growth arrest Mol Cell Biochem 2002 22 2632 2641 10.1128/MCB.22.8.2632-2641.2002 PMC133717 11909957

[B84] Tan NS Shaw NS Vinckenbosch N Liu P Yasmin R Desvergne B Wahli W Noy N Selective cooperation between fatty acid binding proteins and peroxisome proliferator activated receptors in regulating transcription Mol Cell Biochem 2002 22 5114 5127 10.1128/MCB.22.14.5114-5127.2002 PMC139777 12077340

[B85] Gottlicher M Widmark E Li Q Gustafsson JA Fatty acids activate a chimera of the clofibric acid-activated receptor and the glucocorticoid receptor Proc Natl Acad Sci USA 1992 89 4653 4657 10.1073/pnas.89.10.4653 1316614 PMC49141

[B86] Keller H Dreyer C Medin J Mahfoudi A Ozato K Wahli W Fatty acids and retinoids control lipid metabolism through activation of peroxisome proliferator-activated receptorretinoid X receptor heterodimers Proc Natl. Acad Sci USA 1993 90 2160 2164 10.1073/pnas.90.6.2160 8384714 PMC46045

[B87] Lemberger T Desvergne B Wahli W Peroxisome proliferator-activated receptors: a nuclear receptor signalling pathway in lipid physiology Annu Rev Cell Dev Biol 1996 12 335 363 10.1146/annurev.cellbio.12.1.335 8970730

[B88] Desvergne B Wahli W Peroxisome proliferator-activated receptors: nuclear control of metabolism Endocr Rev 1999 20 649 688 10.1210/er.20.5.649 10529898

[B89] Escher P Wahli W Peroxisome proliferator activated receptors: insights into multiple cellular functions Mutat Res 2000 448 121 138 10.1016/S0027-5107(99)00231-6 10725467

[B90] Wolfrum C Borrmann CM Borchers T Spener F Fatty acids and hypolipidemic drugs regulate PPARα and PPARγ gene expression via L-FABP: a signaling path to the nucleus Proc Natl Acad Sci USA 2001 98 2323 2328 10.1073/pnas.051619898 11226238 PMC30137

[B91] Wilk BK Kiec AD Olszanecka A Bodzioch M Kawecka-Jaszcz K The selected pathophysiological aspects of PPARs activation J Physiol Pharmacol 2005 56 149 162 15985699

[B92] Kaikaus RM Chan WK de Ortiz Montellano PR Bass NM Mechanisms of regulation of liver fatty acid-binding protein Mol Cell Biochem 1993 123 93 100 10.1007/BF01076479 8232272

[B93] Wolfrum C Ellinghaus P Fobker M Seedorf U Assmann G Börchers T Spener F Phytanic acid is ligand and transcriptional activator of murine liver fatty acid binding protein J Lipid Res 1999 40 708 714 10191295

[B94] Hansmannel F Clémencet MC Le Jossic-Corcos C Osumi T Latruffe N Nicolas-Francés V Functional characterization of a peroxisome proliferator response-element located in the intron 3 of rat peroxisomal thiolase B gene Biochem Biophys Res Commun 2003 311 149 155 10.1016/j.bbrc.2003.09.185 14575706

[B95] National Research Council Nutrient Requirements of Fish 1993 Washington DC: The National Academies Press

[B96] Goolish EM Okutake K Lesure S Growth and survivorship of larval zebrafish (*Danio rerio*) on processed diets N Am J Aquacult 1999 61 189 198 10.1577/1548-8454(1999)061<0189:GASOLZ>2.0.CO;2

[B97] Meinelt T Schulz C Wirth M Kuerzinger H Steinberg C Dietary fatty acid composition influences the fertilization rate of zebrafish (*Danio rerio*) J Appl Ichthyol 1999 15 19 23 10.1046/j.1439-0426.1999.00121.x

[B98] Bradford Y Conlin T Dunn N Fashena D Frazer K Howe DG Knight J Mani P Martin R Moxon SA Paddock H Pich C Ramachandran S Ruef BJ Ruzicka L Bauer Schaper H Schaper K Shao X Singer A Sprague J Sprunger B Van Slyke C Westerfield M ZFIN: enhancements and updates to the zebrafish model organism database Nucleic Acids Res 2011 39 D822 829 10.1093/nar/gkq1077 21036866 PMC3013679

[B99] Westerfield M The Zebrafish Book: A Guide for the Laboratory Use of Zebrafish (Danio rerio) 2000 Eugene (OR): University of Oregon Press

[B100] Saito Y Tanaka Y Glutaraldehyde fixation of fish tissues for electron microscopy J Electron Microsc 1980 29 1 7

[B101] Bozzola JJ Russell LD Electron Microscopy: Principles and Techniques for Biologists 1999 Sudbury (MA): Jones and Bartlett Publishers

[B102] Luft JH Improvements in epoxy resin embedding methods J Biophys Biochem Cytol 1961 9 409 414 10.1083/jcb.9.2.409 13764136 PMC2224998

[B103] Bustin SA Benes V Nolan T Pfaffl MW Quantitative real-time RT-PCR - a perspective J Mol Endocrinol 2005 34 597 601 10.1677/jme.1.01755 15956331

[B104] Tang R Dodd A Lai D McNabb WC Love DR Validation of zebrafish (*Danio rerio*) reference genes for quantitative real-time RT-PCR normalization Acta Biochim Biophys Sin 2007 39 384 390 10.1111/j.1745-7270.2007.00283.x 17492136 PMC7110012

